# An Intratumor Heterogeneity-Related Signature for Predicting Prognosis, Immune Landscape, and Chemotherapy Response in Colon Adenocarcinoma

**DOI:** 10.3389/fmed.2022.925661

**Published:** 2022-07-07

**Authors:** Cong Liu, Dingwei Liu, Fangfei Wang, Jun Xie, Yang Liu, Huan Wang, Jianfang Rong, Jinliang Xie, Jinyun Wang, Rong Zeng, Feng Zhou, Yong Xie

**Affiliations:** ^1^Department of Gastroenterology, Digestive Disease Hospital, The First Affiliated Hospital of Nanchang University, Nanchang, China; ^2^Gastroenterology Institute of Jiangxi Province, Nanchang, China; ^3^Key Laboratory of Digestive Diseases of Jiangxi Province, Nanchang, China; ^4^Jiangxi Clinical Research Center for Gastroenterology, Nanchang, China

**Keywords:** colon adenocarcinoma, intratumor heterogeneity, prognostic, tumor microenvironment, chemotherapy response

## Abstract

**Background:**

Colon adenocarcinoma (COAD) is a frequent malignancy of the digestive system with a poor prognosis and high mortality rate worldwide. Intratumor heterogeneity (ITH) is associated with tumor progression, poor prognosis, immunosuppression, and therapy resistance. However, the relationship between ITH and prognosis, the immune microenvironment, and the chemotherapy response in COAD patients remains unknown, and this knowledge is urgently needed.

**Methods:**

We obtained clinical information and gene expression data for COAD patients from The Cancer Genome Atlas (TCGA) database. The DEPTH2 algorithm was utilized to evaluate the ITH score. X-tile software was used to determine the optimal cutoff value of the ITH score. The COAD patients were divided into high- and low-ITH groups based on the cutoff value. We analyzed prognosis, tumor mutation burden (TMB), gene mutations, and immune checkpoint expression between the high- and low-ITH groups. Differentially expressed genes (DEGs) in the high- and low-ITH groups were subjected to Gene Ontology (GO) and Kyoto Encyclopedia of Genes and Genomes (KEGG) pathway enrichment analyses. We performed univariate Cox regression and least absolute shrinkage and selection operator (LASSO) regression analyses to screen the prognosis-related genes for the construction of an ITH-related prognostic signature. The nomogram was used to predict the overall survival (OS) of COAD patients. The protein–protein interaction (PPI) network was constructed by using the GeneMANIA database. Principal component analysis (PCA) and single-sample gene set enrichment analysis (ssGSEA) were employed to explore the differences in biological pathway activation status between the high- and low-risk groups. The proportion and type of tumor-infiltrating immune cells were evaluated by the CIBERSORT and ESTIMATE algorithms. Additionally, we assessed the chemotherapy response and predicted small-molecule drugs for treatment. Finally, the expression of the prognosis-related genes was validated by using the UALCAN database and Human Protein Atlas (HPA) database.

**Results:**

The OS of the high-ITH group was worse than that of the low-ITH group. A positive correlation between ITH and TMB was identified. In subgroups stratified by age, gender, and tumor stage, the OS of the low-ITH group remained better than that of the high-ITH group. There were dramatic differences in the mutated genes, single nucleotide variant classes, variant types, immune checkpoints and cooccurring and mutually exclusive mutations of the DEGs between the high- and low-ITH groups. Based on the DEGs between the high- and low-ITH groups, we constructed a five-gene signature consisting of CEACAM5, ENO2, GABBR1, MC1R, and SLC44A4. The COAD patients were divided into high- and low-risk groups according to the median risk score. The OS of the high-risk group was worse than that of the low-risk group. The nomogram was used to accurately predict the 1-, 3- and 5-year OS of COAD patients and showed good calibration and moderate discrimination ability. The stromal score, immune score, and ESTIMATE score of the high-risk group were significantly higher than those of the low-risk group, whereas tumor purity showed the opposite trend. The patients classified by the risk score had distinguishable sensitivity to chemotherapeutic drugs. Finally, two public databases confirmed that CEACAM5 and SLC44A4 were upregulated in normal tissues compared with COAD tissues, and ENO2, GABBR1, and MC1R were upregulated in COAD tissues compared with normal tissues.

**Conclusion:**

Overall, we identified an ITH-related prognostic signature for COAD that was closely related to the tumor microenvironment and chemotherapy response. This signature may help clinicians make more personalized and precise treatment decisions for COAD patients.

## Introduction

Colon adenocarcinoma (COAD), a highly heterogeneous malignancy, is the third most common form of cancer and the second leading cause of cancer-related death worldwide (1, 2). Most COAD cases are diagnosed in an advanced stage due to its insidious onset, delayed diagnosis, and proclivity for metastasis, which contribute to a poor prognosis. Despite advances in current standard treatment strategies, such as surgery, chemotherapy, radiotherapy, and immunotherapy, the 5-year OS of COAD patients remains dismal (3). Therefore, there is an urgent need to develop a novel biomarker to improve outcomes in patients with COAD, allowing early intervention and reducing the increasing burden of COAD.

One of the characteristics of COAD is intratumoral and individual-level heterogeneity heterogeneity (4). Intratumor heterogeneity (ITH) refers to differences in molecular and phenotypic profiles between different tumor cells within a tumor (5). The available evidence suggests that ITH is strongly associated with tumor progression, aggressiveness, treatment resistance, and disease recurrence in various cancer types (6, 7). ITH may increase tumor resistance to therapy and act as an independent prognostic factor for COAD (8). With the rise of next-generation sequencing technology and machine learning, researchers have developed various methods (MATH, PhyloWGS, ABSOLUTE and tITH) for quantifying ITH (9–12). Song et al. showed that DEPTH2 is a novel algorithm for evaluating ITH based on mRNA levels, and compared with other algorithms, DEPTH2 has wider application and stronger competitiveness in characterizing ITH (13).

In the present study, we quantified the ITH of COAD in patients from the TCGA database by using the DEPTH2 algorithm. We analyzed prognosis, tumor mutational burden (TMB), mutated genes, and immune checkpoint expression in the high- and low-ITH groups. Next, we constructed an ITH-related signature and separated COAD patients into high- and low-risk groups based on the median risk score. In addition, we explored the association of the signature with the prognosis, immune microenvironment, and chemotherapy response and further validated the predictive value of the signature. Collectively, our findings indicate that the ITH-related signature is a robust and reliable biomarker that may provide new insights into the prognosis and treatment of COAD.

## Materials and Methods

### Data Collection and Processing

RNA sequencing transcriptome data, somatic mutation data, and the corresponding clinical information of COAD patients were downloaded from the TCGA database (https://portal.gdc.cancer.gov/). The detailed clinical information for the COAD patients is presented in Supplementary Table S1. Patients were excluded if they had incomplete survival information; in all, 452 COAD patients were assessed.

### Calculation of the ITH Score and Definition of the Optimal Cutoff Value

DEPTH2 is a new algorithm for assessing ITH at the mRNA level; it has broad applications and is highly competitive with other algorithms for ITH measurement. Here, we calculated the ITH score using the DEPTH2 algorithm (13). The principle of X-tile software (version 3.6.1, Yale University, USA) was to group different ITH scores to determine cutoff values for statistical analysis, and the result with *p* < 0.05 was the optimal cutoff value for separating patients into high- and low-ITH groups (14).

### Gene Mutation Analysis

Somatic mutations of the top twenty mutated genes between the high- and low-risk groups were presented using the “maftools” package. Then, we calculated the TMB for each patient in the high- and low-risk groups. Additionally, the correlation between the TMB and ITH score was evaluated via Spearman correlation analysis.

### Identification of Differentially Expressed ITH-Related Genes

The differentially expressed genes (DEGs) between the high- and low-ITH groups were analyzed using the “limma” package, with the filter criteria of |log2Fold change (FC) | > 0.5 and *p*-value < 0.05. The DEGs were subjected to Gene Ontology (GO) and Kyoto Encyclopedia of Genes and Genomes (KEGG) pathway enrichment analyses with the “clusterProfiler” package.

### Construction and Validation of the ITH-Related Prognostic Signature

The DEGs were subjected to univariate Cox regression analysis to acquire genes related to OS. Least absolute shrinkage and selection operator (LASSO) Cox regression analysis was conducted using the “glmnet” package. Then, we performed multivariate Cox regression analysis and identified five genes. The ITH-related prognostic signature score was calculated based on the following formula: Risk score = (−0.15233956) × CEACAM5 + (0.113295033) × ENO2 + (0.159639519) × GABBR1 + (0.5952466) × MC1R + (0.039993577) × SLC44A4.

The patients were classified into high- or low-risk groups according to the median risk score. We conducted Kaplan–Meier (KM) survival analysis and compared the overall survival (OS) between the two subgroups to assess the prognostic value of the signature. Time-dependent receiver operating characteristic (ROC) curves were generated using the “timeROC” package. To analyze the impact of the ITH-related prognostic signature on COAD progression, we clarified the relationship between the risk score and clinical parameters (gender, age, and stage) with the chi-square test.

### Nomogram Establishment and Assessment

The predictive nomogram combining the risk score and clinical features was constructed by using the “rms” and “regplot” packages. We evaluated the prognosis of COAD patients at 1, 3, and 5 years in the nomogram. Calibration curves, ROC curves, and decision curve analysis (DCA) were applied to verify the reliability and accuracy of the nomogram.

### Functional Enrichment Analysis

The correlation of the five key prognostic genes was conducted by Spearman correlation analysis. GeneMANIA (http://www.genemania.org) is an interactive, user-friendly, and intuitive website that constructs a protein–protein interaction (PPI) network (15, 16). In the present study, a PPI network for five key prognostic genes was constructed by using the GeneMANIA website. Principal component analysis (PCA) was utilized to assess the classification accuracy of the signature. Hallmark gene sets between the high- and low-risk groups were analyzed by using the ssGSEA algorithm. The mRNA expression levels of crucial prognostic genes were acquired from the UALCAN database (http://ualcan.path.uab.edu/). The protein expression levels of crucial prognostic genes were obtained based on immunohistochemical (IHC) staining images downloaded from the Human Protein Atlas database (HPA; https://www.proteinatlas.org/).

### Evaluation of Immune Cell Infiltration

The infiltration levels of 22 tumor-infiltrating immune cells were evaluated by using the CIBERSORT algorithm. We implemented the ESTIMATE algorithm to calculate the stromal score, immune score, ESTIMATE score and tumor purity. Single-sample gene set enrichment analysis (ssGSEA) was performed to quantify the immune enrichment score of each sample. The anticancer immune response, also known as the cancer–immunity cycle, is characterized by seven steps that occur in the tumor microenvironment. Tracking Tumor Immunophenotype (TIP; http://biocc.hrbmu.edu.cn/TIP/) is a platform for evaluating and visualizing antitumor immune activity and the proportion of tumor-infiltrating immune cells during each of the seven steps (17). Here, we analyzed the anticancer immune status and extent of tumor-infiltrating immune cells in the high- and low-risk groups as well as differences in the activity scores across the seven steps.

### Chemotherapeutic Sensitivity and Small-Molecule Drugs

The chemotherapeutic drug response of the COAD patients was evaluated by using the Genomics of Drug Sensitivity in Cancer (GSDC) database (https://www.cancerrxgene.org/) (18). We calculated the half-maximal inhibitory concentration (IC50) using the “pRRophetic” package and assessed the patient response to common chemotherapeutic agents. In addition, we uploaded DEGs to the Connectivity Map (cMap; https://portals.broadinstitute.org/cmap/) database and predicted drugs based on the prognostic signature. Enrichment scores ranged from −1 to 0, and *p* < 0.05 was considered to indicate a potential candidate compound. The 3D structures of these compounds were obtained from the PubChem database (https://pubchem.ncbi.nlm.nih.gov/).

### Statistical Analysis

R software (Version 4.0.5, https://www.r-project.org/) was used for all statistical analyses and plotting. Correlation was performed using Spearman's correlation. A Cox regression model was performed for univariate and multivariate analyses. Differences in clinical characteristics between the different groups were analyzed with the chi-square test. *p* < 0.05 was considered to indicate statistical significance.

## Results

### ITH Estimation and Stratification Analysis in COAD Patients

**Figure 1:**
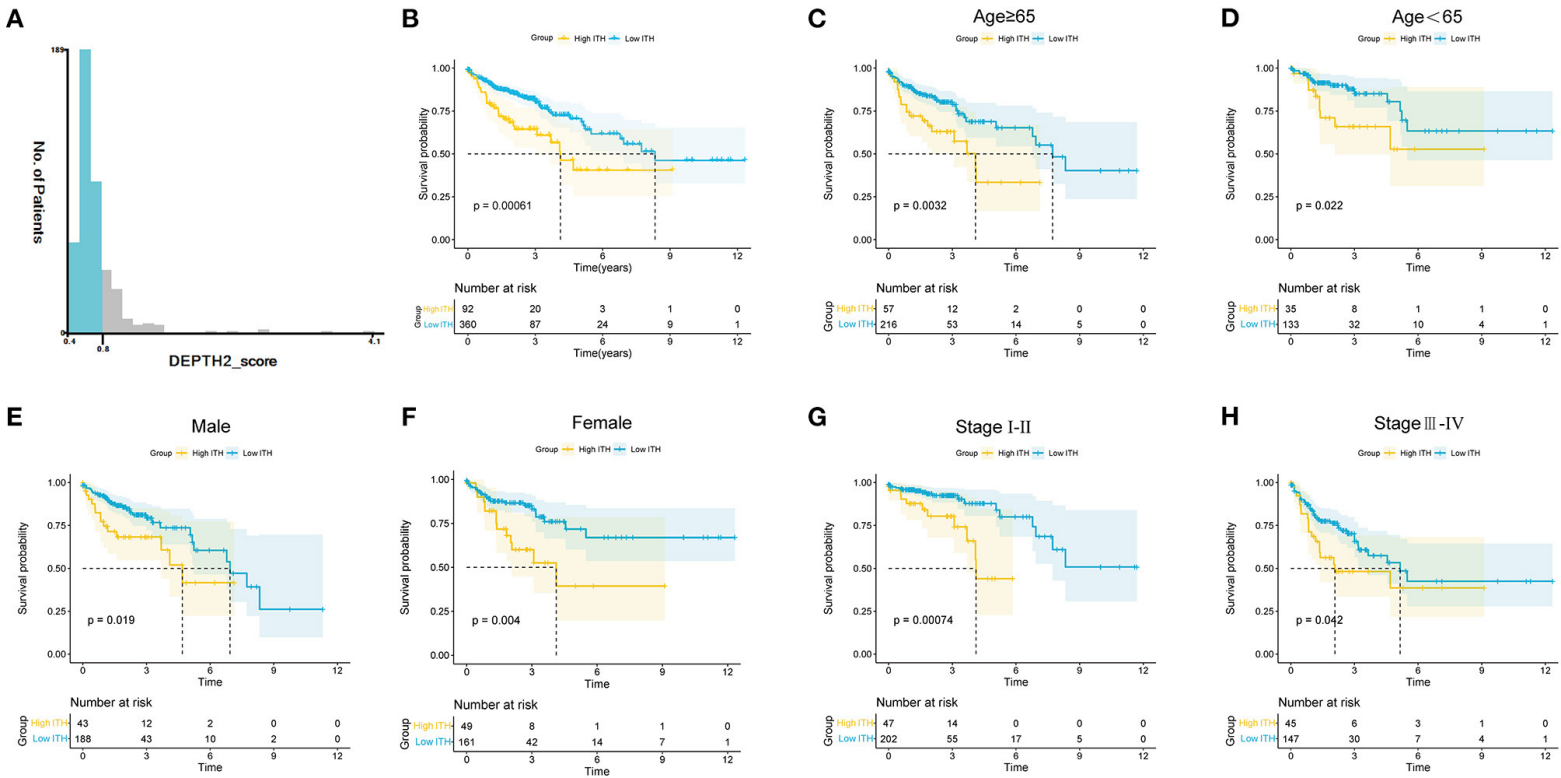
ITH estimation and stratification analysis of COAD patients. **(A)** COAD patients were divided into two groups with X-tile software. **(B)** KM survival curves of the OS for the COAD patients in the high- and low-ITH groups. **(C,D)** KM survival curves of COAD patients age>65 **(C)** and age <65 **(D)**. **(E,F)** KM survival curves of male **(E)** and female **(F)** COAD patients. **(G,H)** KM survival curves of COAD patients with stage I-II **(G)** and stage III-IV **(H)**.

The flow diagram of this study is shown in Supplementary Figure S1. First, we calculated the ITH score of 452 COAD patients from the TCGA database using the DEPTH2 algorithm. Then, we used X-tile software to determine that the optimal cutoff value for the ITH score was 0.78 (Figure 1A). The COAD patients were segregated into high- and low-ITH groups based on the cutoff value. The high-ITH group had poorer OS than the low-ITH group (Figure 1B). Furthermore, in subgroups stratified by age, gender, and tumor stage, the low-ITH group had better OS than the high-ITH group (Figures 1C–H).

### Mutations and Immunotherapeutic Responses of the two Subgroups

**Figure 2:**
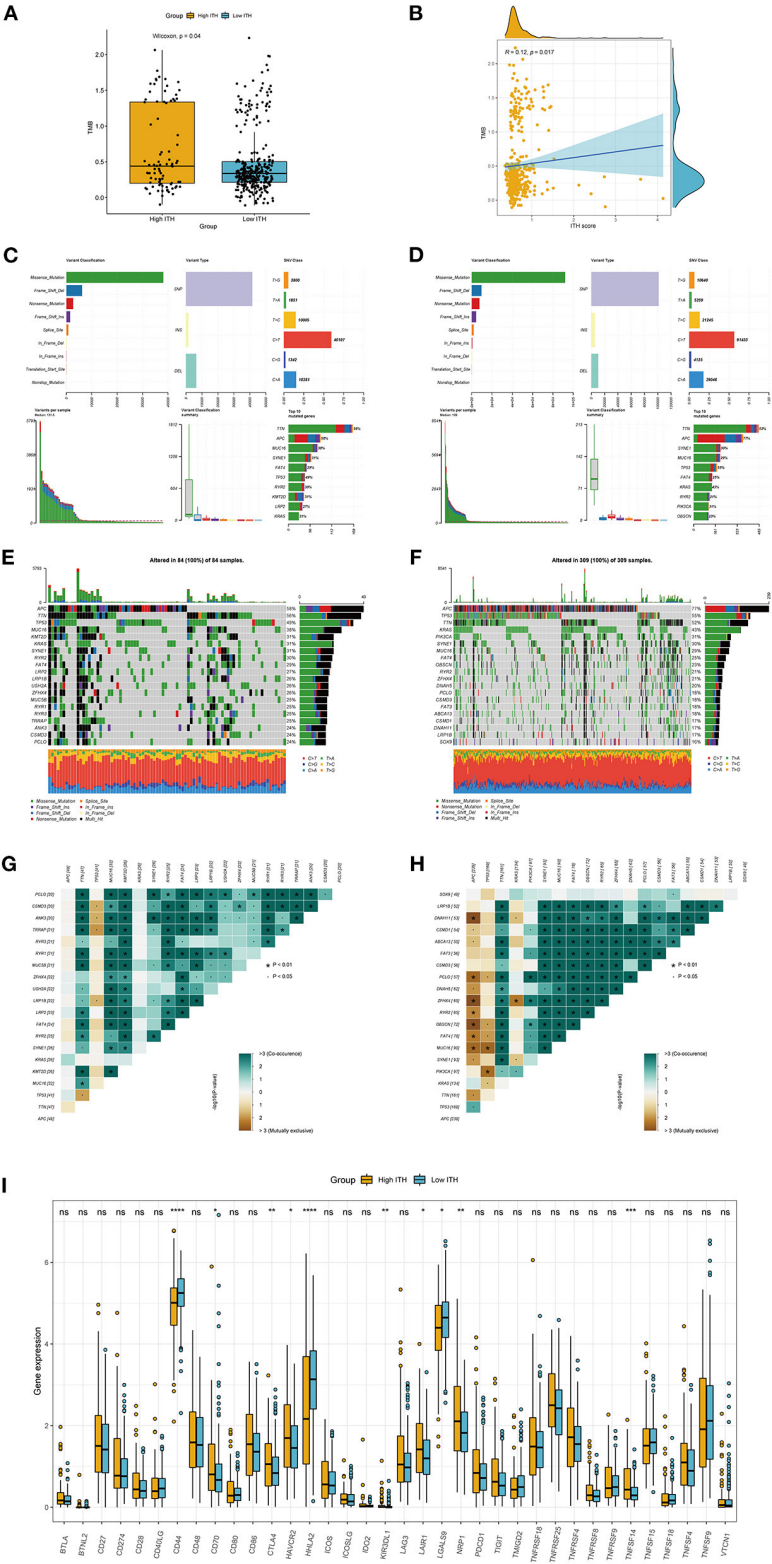
TMB and immunotherapeutic responses in the high- and low-ITH groups. **(A)** Comparison of the TMB between the high- and low-risk groups. **(B)** The association between the TMB and ITH score. **(C,D)** The distribution of different types of mutations in the high-ITH **(C)** and low-ITH groups **(D)**. The upper panel shows the variant classification, variant type, and single nucleotide variant class of mutated genes, and the lower panel shows the variants per sample, variant classification summary, and top ten mutated genes. **(E,F)** Waterfall plot of somatic mutation features in the high-ITH **(E)** and low-ITH groups **(F)**. The middle panel represents the gene mutation patterns for each sample. The upper bar plot represents the total mutation burden for each patient. The right panel depicts the mutation frequencies for each gene. **(G,H)** The co-occurring and mutually exclusive mutations among the differentially mutated genes in the high-ITH **(G)** and low-ITH groups **(H)**. **(I)** The expression levels of immune checkpoint genes between the high- and low-ITH groups.

Considering that the TMB is associated with the efficacy of immunotherapy, we calculated the TMB for each COAD patient. As expected, the high-TMB group tended to have a higher TMB than the low-TMB group (Figure 2A). Furthermore, we evaluated the latent relevance of ITH and TMB in COAD patients. As shown in Figure 2B, there was a positive correlation between ITH and TMB via Spearman correlation analysis (*r* = 0.12, *p* = 0.017, Figure 2B). Figures 2C,D summarize the mutation information for the high- and low-ITH groups, respectively. Subsequently, the mutations associated with the different ITH subgroups were visualized by waterfall plots. APC (58%), TTN (56%), TP53 (49%), MUC16 (38%) and KMT2D (31%) were the top five genes with the highest mutation frequencies in the high-ITH group, while APC (77%), TP53 (55%), TTN (52%), KRAS (43%) and PIK3CA (31%) were the top five genes in the low-ITH group (Figures 2E,F). In addition, we observed the co-occurrence of mutations, such as TTN and KMT2D mutations, in the high-ITH group (Figure 2G) and mutually exclusive mutations, such as APC and DNAH11 mutations, in the low-ITH group (Figure 2H). Given the importance of immune checkpoint inhibitors in the clinical treatment of COAD, we further analyzed the differential expression of immune checkpoint genes and found significant differences in the levels of CD44, CD70, CTLA4, HAVCR2, and HHLA2 between the high- and low-ITH groups (Figure 2I).

### Identification of Differentially Expressed ITH-Related Genes

**Figure 3:**
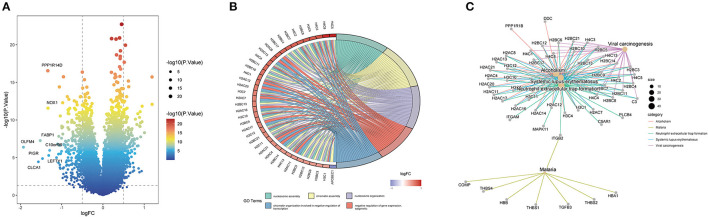
Identification and enrichment analysis of ITH-related genes in COAD patients. **(A)** Volcano plot of the ITH-related DEGs. **(B)** GO annotation of the DEGs. **(C)** KEGG pathway analysis of the DEGs.

Through differential gene expression analysis between the high- and low-ITH groups, we obtained 421 differentially expressed ITH-related genes, namely, 173 upregulated genes and 248 downregulated genes. These DEGs were visualized using a volcano plot (Figure 3A). The 421 DEGs were further subjected to functional enrichment analysis. The top five significant GO terms associated with the DEGs were “nucleosome assembly,” “chromatin assembly,” “nucleosome organization,” “chromatin organization involved in negative regulation of transcription,” and “negative regulation of gene expression, epigenetic” (Figure 3B). The KEGG pathway enrichment analysis indicated that these DEGs were related to alcoholism, malaria, neutrophil extracellular trap formation, systemic lupus erythematosus, and viral carcinogenesis (Figure 3C).

### Construction and Validation of an ITH-Related Prognostic Signature

**Figure 4:**
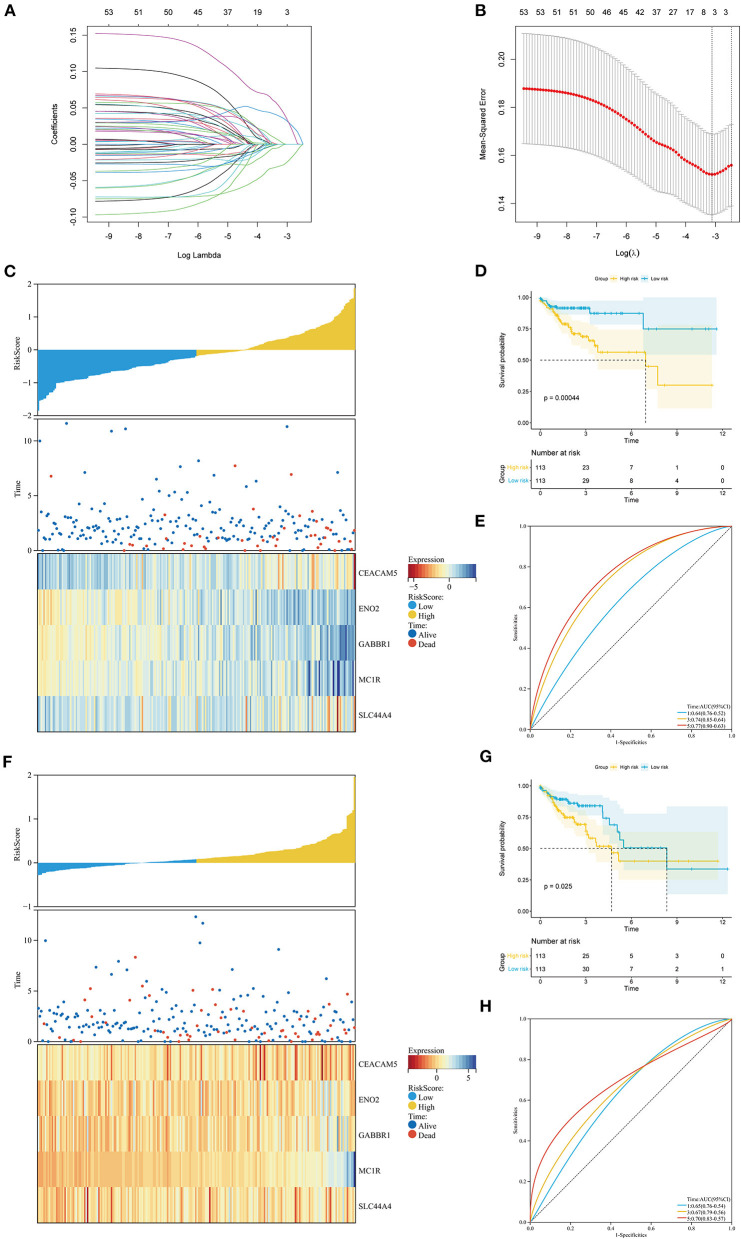
Construction of the ITH-related prognostic signature in the training cohort and the validation cohort. **(A)** The LASSO coefficient profile of 53 prognostic ITH-related genes. **(B)** Identification of the optimal tuning parameter (log λ) LASSO model using cross-validation. The abscissa axis is log λ, and the vertical axis is the mean squared error. **(C,F)** The distribution plot of the risk score (upper), survival time (middle), and heatmap for the expression of five genes (below) in the training cohort **(C)** and the validation cohort **(F)**. **(D,G)** KM survival analysis of OS between the high- and low-risk groups in the training cohort **(D)** and the validation cohort **(G)**. **(E,H)** ROC curve analysis of the ITH-related prognostic signature for predicting the 1-, 3-, and 5-year OS of COAD patients in the training cohort **(E)** and the validation cohort **(H)**.

Based on the ITH-related genes, we constructed an ITH-related prognostic signature. First, we identified 53 survival-related genes by using univariate Cox regression analysis. Then, five ITH-related prognostic genes were further screened through LASSO Cox regression analysis (Figures 4A,B). The COAD patients were randomly divided into a training cohort (*n* = 226) or a validation cohort (*n* = 226) at a ratio of 1:1. Then, we calculated the risk score of each patient, and the COAD patients were separated into high- and low-risk groups based on the median risk score. The distribution plot of the risk scores for the high- and low-risk groups is shown in Figure 4C. The survival times for the COAD patients are displayed in Figure 4C. The heatmap showed the expression profiles of five crucial genes in the high- and low-risk groups (Figure 4C). In addition, the KM survival curves indicated that the survival time for the low-risk group was markedly longer than that for the high-risk group (Figure 4D). The areas under the ROC curves (AUCs) for 1-, 3-, and 5-year survival were 0.64, 0.74, and 0.77, respectively, which revealed that the ITH-related prognostic signature might effectively predict the outcomes of patients with COAD (Figure 4E).

To further validate the universality of the prognostic signature, we conducted a similar analysis in the validation cohort and the entire cohort. The risk score for each patient was calculated using the same formula. The distribution of the risk scores, survival times of the patients, and ITH-related prognostic gene expression profiles are shown in Figure 4F and Supplementary Figure S2A. The high-risk group had a poorer prognosis than the low-risk group in both the validation cohort and the entire cohort (Figure 4G; Supplementary Figure S2B). The AUCs of the 1-, 3-, and 5-year survival were 0.65, 0.67, and 0.70 in the validation cohort and 0.63, 0.67, and 0.68 in the entire cohort (Figure 4H; Supplementary Figure S2C).

### Correlation Between the Prognostic Signature and Clinical Features

**Figure 5:**
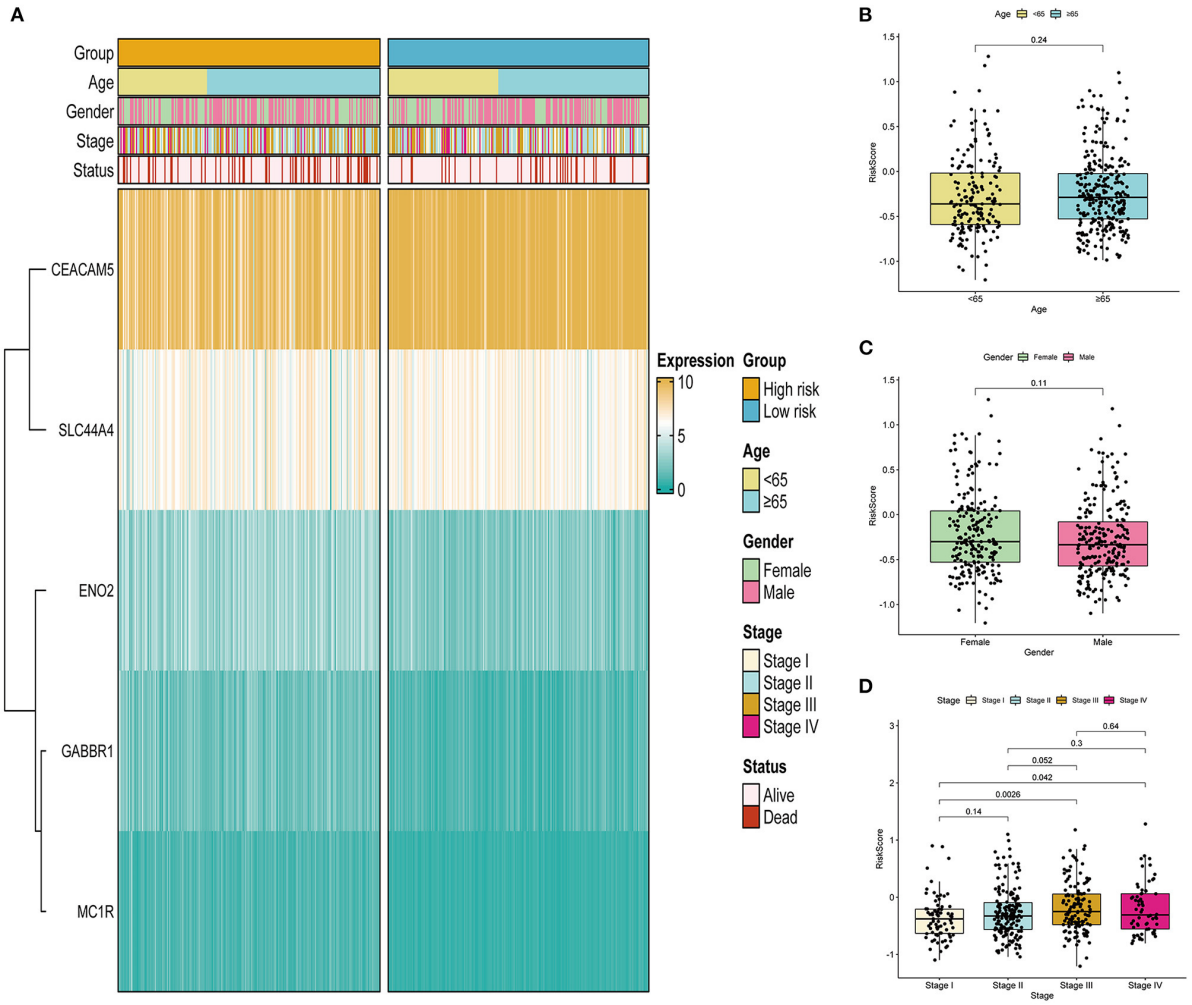
Correlation between the ITH-related prognostic signature and clinical features in the TCGA cohort. **(A)** Heatmap of the association between the expression levels of the five genes and clinical features. **(B–D)** Correlation analysis of the ITH-related prognostic signature with age **(B)**, gender **(C)**, and stage **(D)**.

As displayed in Figure 5A, we analyzed the associations between the risk scores and clinical features, such as age, gender, and stage. We found that the expression level of CEACAM5 decreased with increasing risk score, whereas the expression level of MC1R increased with increasing risk score. We further analyzed the value of the risk score between the two groups stratified by different clinical features. Figure 5B illustrates that the correlation of age and risk score was not statistically significant (*p* = 0.24). As shown in Figure 5C, our results indicated that the correlation of gender and risk score was not statistically significant (*p* = 0.11). In addition, Figure 5D shows that the risk scores were significantly different between the stage I and stage III groups (*p* < 0.05) and between the stage I and stage IV groups (*p* < 0.05). In brief, the risk scores different among different stage groups, and the association of stage and risk score was statistically significant.

### Construction of a Clinical Nomogram for Predicting Survival

**Figure 6:**
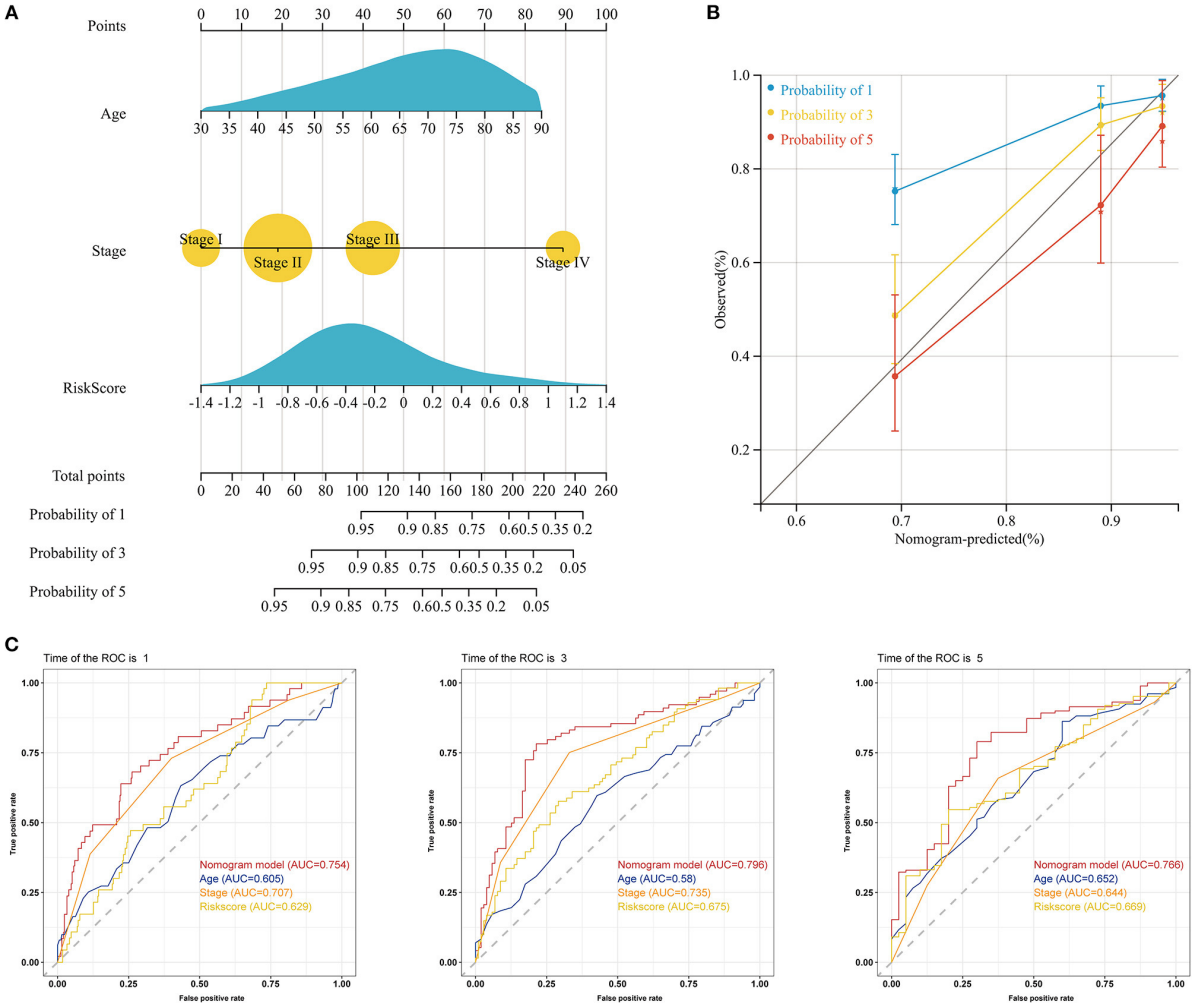
Construction of a nomogram predicting the OS of patients with COAD in the TCGA cohort. **(A)** Nomogram for predicting the 1-, 3-, and 5-year OS of COAD patients. **(B)** Calibration plots of the nomogram for predicting the 1-, 3-, and 5-year OS of COAD patients. The X-axis represents the nomogram-predicted survival, and the Y-axis represents the actual survival. **(C)** The time-dependent ROC curves of the nomogram for predicting the 1-, 3-, and 5-year OS of COAD patients.

To determine whether the prognostic signature was an independent prognostic factor for COAD, we performed univariate and multivariate Cox regression analyses (Supplementary Figures S3A,B). Univariate Cox regression analysis showed that the risk score was highly correlated with OS [hazard ratio (HR): 2.75, 95% CI: 1.81–4.20, *p* < 0.001]. Moreover, multivariate Cox regression analysis further indicated that the risk score could be used to independently predict the prognosis of COAD patients (HR: 2.21, 95% CI: 1.45–3.36, *p* < 0.001). Subsequently, our prognostic nomogram integrating the risk score, age, and stage was constructed to estimate the 1-, 3-, and 5-year OS for COAD patients (Figure 6A). The calibration curve indicated that the actual observation vs. prediction rates for 1-, 3- and 5-year OS demonstrated excellent consistency (Figure 6B). The AUCs of the nomogram for predicting 1-, 3-, and 5-year OS were 0.754, 0.796, and 0.766, respectively, which were higher than the values for any of the individual factors (Figure 6C). Moreover, the DCA curves indicated that the clinical nomogram with diverse clinical features provided more net benefits in predicting the prognosis of COAD patients than any individual factor (Supplementary Figure S3C).

### Functional Analysis of the Prognostic Signature

**Figure 7:**
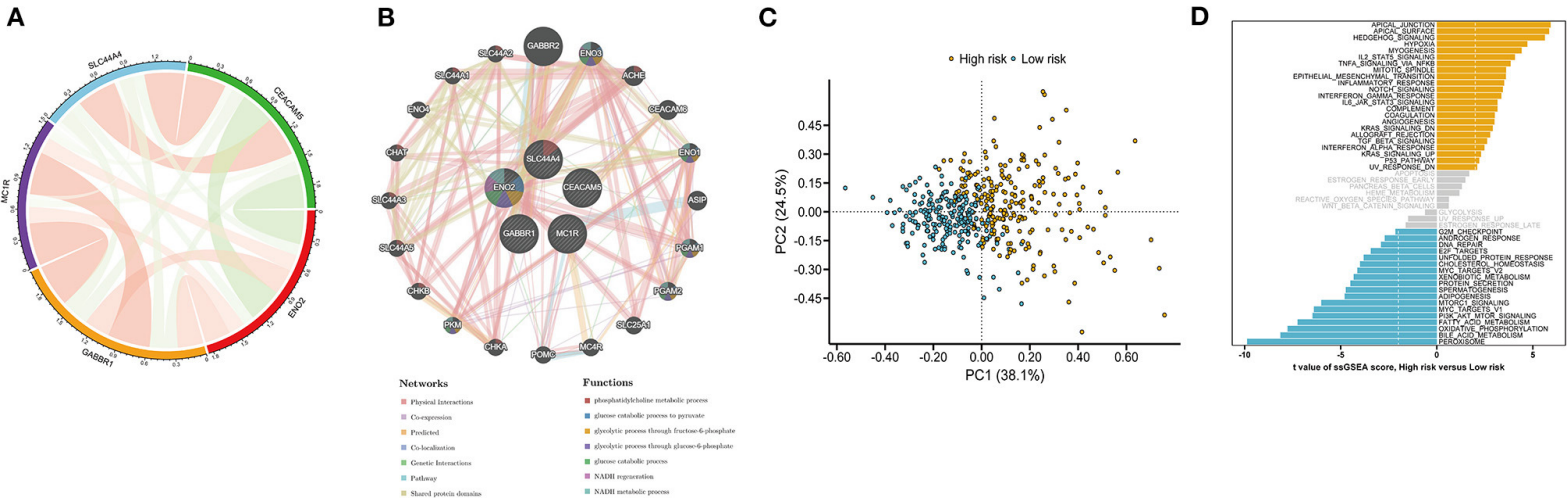
Functional analysis of the DEGs in the high- and low-risk groups. **(A)** The correlation analysis of five key prognostic genes. The green and red lines represent negative and positive correlations, respectively. Line thickness indicates a positive correlation with the strength. **(B)** A protein–protein interaction (PPI) network for five key prognostic genes was constructed by using the GeneMANIA website. Each node represents a gene. Different network edge colors represent the possible functions of the corresponding gene. **(C)** PCA between the high- and low-risk groups. **(D)** Hallmark gene set in the high- and low-risk groups.

Analysis of the correlations among the five key prognostic genes was performed by Spearman correlation analysis (Figure 7A). Our results showed that CEACAM5 was negatively correlated with ENO2, whereas GABBR1 was positively associated with MC1R. Then, a gene–gene interaction network for the five key prognostic genes was constructed by using the GeneMANIA website. The function of these genes was primarily associated with the phosphatidylcholine metabolic process, the glucose catabolic process, NADH regeneration, and the NADH metabolic process (Figure 7B). PCA indicated a clear distribution between the high- and low-risk groups (Figure 7C). To explore the molecular mechanisms involved in the ITH-related prognostic signature, ssGSEA was used to analyze the high- and low-risk groups. The enrichment results indicated that the hallmark genes of the apical junction, apical surface, and hedgehog signaling were activated in the high-risk group, whereas the hallmark genes of oxidative phosphorylation, bile acid metabolism, and peroxisome were activated in the low-risk group (Figure 7D).

### Interrelationship Between the ITH-Related Prognostic Signature and Immune Cell Infiltration

**Figure 8:**
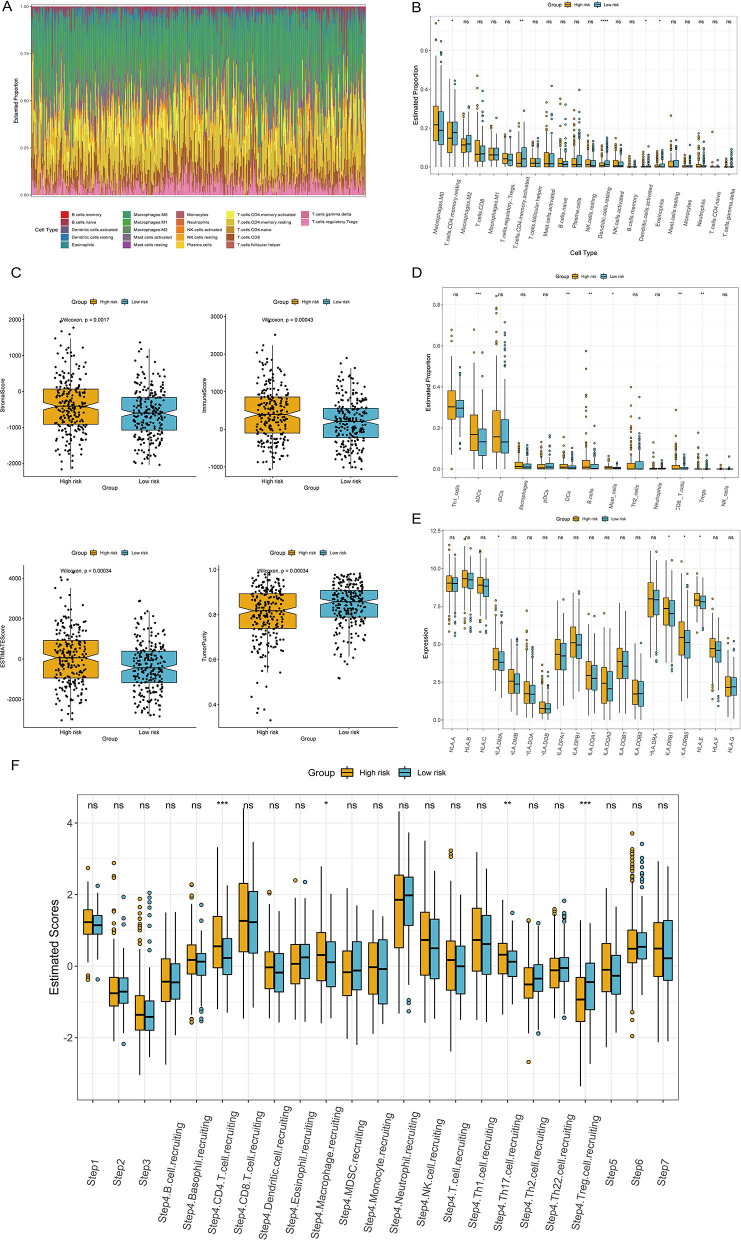
Correlation of the ITH-related prognostic signature with the immune landscape of patients with COAD. **(A)** Heatmap of the proportion of immune cells in each COAD sample. **(B)** The differences in immune cell subtypes between the high- and low-risk groups. **(C)** Correlations between the ITH-related prognostic signature and the stromal score, immune score, ESTIMATE score, and tumor purity. **(D)** The differences in immune cell infiltration in the high- and low-risk groups. **(E)** The differences in the expression of MHC molecules between the high- and low-risk groups. **(F)** Comparison of the relative abundance levels of antitumor immune cells between the high- and low-risk groups.

We comprehensively analyzed the correlation between the ITH-related prognostic signature and 22 tumor-infiltrating immune cells using the CIBERSORT algorithm. The immune landscape of the TCGA-COAD samples is shown in Figure 8A. By comparing and analyzing the immune cell profiles, we observed that M0 macrophages were more highly infiltrated in the high-risk group. Conversely, resting memory CD4+ T cells, activated memory CD4+ T cells, and resting dendritic cells were significantly higher in the low-risk group than in the high-risk group (Figure 8B). In addition, the low-risk group had a lower stromal score, immune score, and ESTIMATE score but a higher tumor purity score than the high-risk group (Figure 8C). We applied the ssGSEA algorithm to the RNA sequencing data for the COAD samples to evaluate immune cell infiltration and related functions. Our results showed that the populations of activated dendritic cells (aDCs), B cells, and dendritic cells (DCs) were higher in the high-risk group (Figure 8D). Moreover, we analyzed the expression of MHC molecules and found that they were higher in the high-risk group (Figure 8E). An antitumor immune response must initiate a cascade of stepwise events to effectively eliminate cancer cells (19). To further analyze the impact of immune cells on COAD, the immune activity score of each step was calculated by RNA expression data and TIP analysis. Then, we compared the differences in the scores for the seven steps between the high- and low-risk groups. The results indicated that the abundance of antitumor immune cells was lower in the low-risk group than in the high-risk group (Figure 8F).

### Analysis of the Chemotherapy Response and Screening of Small-Molecule Drugs

**Figure 9:**
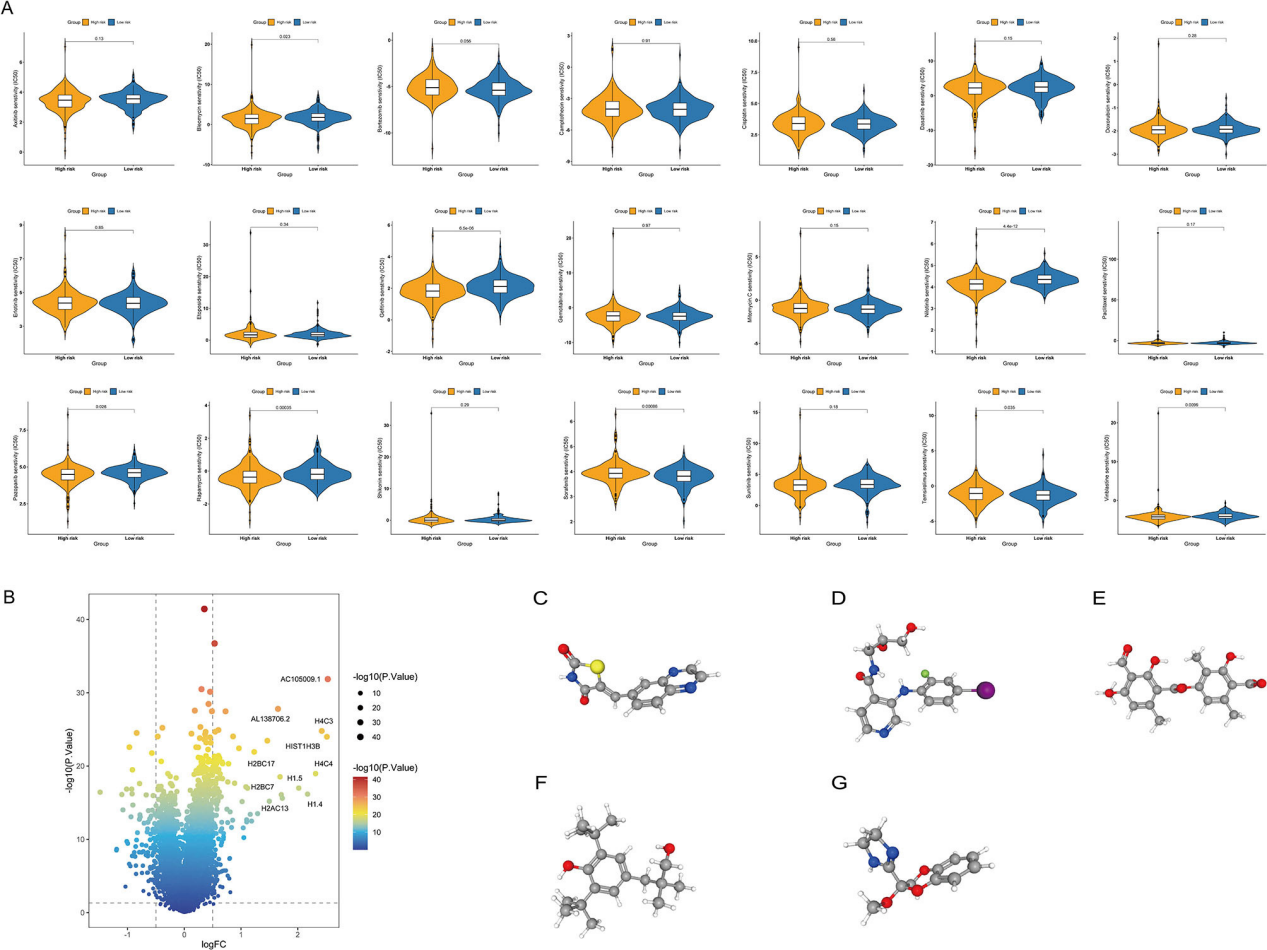
Relationship between the ITH-related prognostic signature and chemotherapy response. **(A)** Differences in the therapeutic sensitivity of common chemotherapy drugs between the high- and low-risk groups. **(B)** Volcano plot of DEGs in the high- and low-risk groups. **(C–G)** The 3D structure of five potential compounds, including AS-605240 **(C)**, AS-703026 **(D)**, baeomycesic acid **(E)**, CGP-7930 **(F)**, and RX-821002 **(G)**.

To determine the efficacy of the ITH-related prognostic signature as a biomarker to predict the therapeutic response in COAD patients, we estimated the IC50 values of common anticancer drug agents between the high- and low-risk groups using the pRRophetic algorithm (Figure 9A). The sensitivity to many chemotherapeutic drugs differed significantly between the high- and low-risk groups (*p* = 0.023 for bleomycin, *p* < 0.001 for gefitinib, p<0.001 for nilotinib, *p* = 0.026 for pazopanib, *p* = 0.00035 for rapamycin, *p* = 0.00086 for sorafenib, *p* = 0.035 for temsirolimus, *p* = 0.0099 for vinblastine).

In addition, we screened potential small-molecule drugs for COAD using the cMap database. By analyzing the DEGs between the low- and high-risk groups, we identified 138 upregulated and 94 downregulated genes (Figure 9B). Based on these DEGs, five potential compounds, namely, AS-605240 (Figure 9C), AS-703026 (Figure 9D), baeomycesic acid (Figure 9E), CGP-7930 (Figure 9F), and RX-821002 (Figure 9G), were screened as potential targeted drug candidates for COAD patients.

### Expression of Prognostic Genes

**Figure 10:**
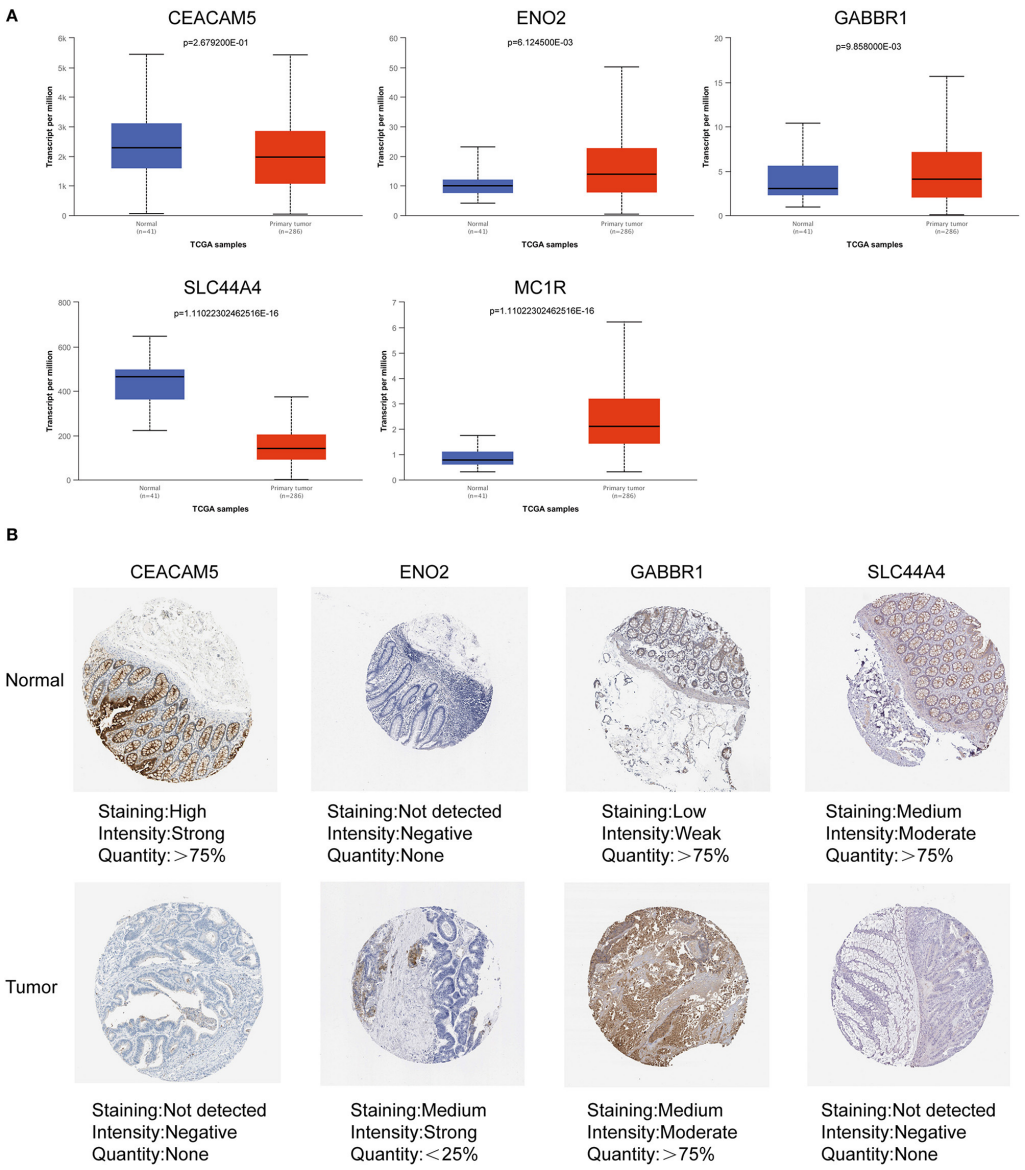
Expression of ITH-related prognostic genes in COAD tissues and normal tissues. **(A)** The mRNA expression levels of prognostic genes in COAD tissues and normal tissues based on the UALCAN database. **(B)** The protein expression levels of prognostic genes in COAD tissues and normal tissues based on the HPA database.

We obtained the mRNA and protein expression levels of five key prognostic genes based on the UALCAN database and HPA database. In Figure 10A, we observed that the mRNA expression levels of CEACAM5 and SLC44A4 were significantly elevated in normal tissues compared with tumor tissues. In contrast, the levels of ENO2, GABBR1, and MC1R were significantly elevated in tumor tissues compared with normal tissues. Similarly, IHC confirmed that the protein expression levels of CEACAM5, ENO2, GABBR1, and SLC44A4 had the same tendency (Figure 10B).

## Discussion

COAD is one of the most malignant tumors in the world, causing more than 850 000 deaths annually (20). Improving the prognosis of COAD is clinically challenging due to its high heterogeneity and metastatic potential (21). ITH endows tumors with multiple abilities and biological properties that make them more prone to metastasis, recurrence, and drug resistance and is therefore considered one of the key reasons for cancer treatment failure (8, 22). A recent study extensively characterized ITH in whole-genome sequences of 2,658 cancer samples across 38 cancer types and revealed some underlying drivers (5). However, a comprehensive understanding of ITH requires more research. Although the role of ITH in the initiation of COAD has been well documented, the extent to which ITH influences the prognosis and treatment strategies for COAD patients remains unclear. In this study, we conducted a comprehensive analysis of the prognosis, immune microenvironment, and chemotherapy response of COAD patients based on the ITH score. We constructed an ITH-related prognostic signature based on the ITH score. Our results indicated that the ITH-related prognostic signature is an independent prognostic factor for predicting the OS of COAD patients. Furthermore, we showed that the ITH-related prognostic signature is valuable for predicting the immune microenvironment and chemotherapy response in COAD.

In the current study, our prognostic signature consisted of five ITH-related genes, namely, CEACAM5, ENO2, GABBR1, SLC44A4, and MC1R. The five-gene signature has good performance in prognosis prediction. It has been reported that these five key prognostic genes are related to tumorigenesis and cancer progression. CEACAM5 is a cell-surface glycoprotein that is normally expressed on epithelial cells in numerous organ systems (23). The high expression of CEACAM5 in colorectal cancer cells is closely associated with the CD133-positive colorectal cancer stem cell phenotype (24). ENO2 functions not only as a glycolysis enzyme but also as a critical activator of other oncogenic pathways (25, 26). The low expression level of ENO2 in colon cancer is associated with a good prognosis (27). GABBR1 is a seven-transmembrane receptor that is localized on chromosome 6p21.3 within the HLA class I region close to the HLA-F gene (28). MiR-106a/b, miR-20a/b, and miR-17 have been reported to be involved in the invasion and proliferation of colorectal cancer by targeting their common target gene GABBR1 (29). SLC44A4 is a member of the family of solute carrier proteins, which are normally expressed on the apical surface of secretory epithelial cells (30). The latest research indicates that SLC44A4 may be a potential target for the diagnosis, prognosis, and treatment of clear cell renal cell carcinoma (31). MC1R has been relevant to an increased susceptibility to skin cancer (32). In addition, MC1R antagonists and agonists might be employed as potential therapeutic drugs for skin cancer (33, 34). MC1R plays a pivotal role in the progression of colorectal cancer and may serve as a marker of worse prognosis in colorectal cancer (35). Accordingly, our prognostic signature may have the potential to be a novel biomarker and provide new insights into the mechanisms of COAD from the prospect of ITH.

We used X-tile software to obtain the optimal cutoff value of the ITH score and divided the COAD patients into high- and low-ITH groups. The OS of patients in the high-ITH group was worse than that of patients in the low-ITH group. Subsequently, we performed subgroup analysis by age, gender and stage and found that the patients in the low-ITH group had better OS than those in the high-ITH group. In addition, the patients in the low-risk group had better OS than those in the high-risk group in the training cohort, validation cohort, and entire cohort. The AUC values for 3-year survival in the training cohort and validation cohort were 0.74 and 0.67, respectively, indicating that the model has better predictive ability than similar prognostic models in other studies (AUC = 0.63, AUC = 0.585) (36, 37). Then, we identified the risk score as an independent prognostic factor by univariate and multivariate Cox regression analyses. We constructed a nomogram by integrating the risk score, age, and stage. The results confirmed the stability and reliability of the nomogram in predicting 1-, 3-, and 5-year OS in COAD patients. In brief, the AUC value of the nomogram was significantly better than that of other clinical factors, indicating that the nomogram can guide clinicians in the diagnosis and treatment of COAD.

The tumor microenvironment is composed of tumor cells, various infiltrating immune cells, stromal cells, and cytokines (38). With the continuous in-depth study of the tumor microenvironment, the important role of infiltrating immune cells in the occurrence, development, metastasis, recurrence, and immune escape of COAD has been highlighted (39). Therefore, infiltrating immune cells may be a promising therapeutic target (40). In this study, we analyzed the types and proportions of infiltrating immune cells in the high- and low-risk groups through the CIBERSORT algorithm. Our results showed that resting memory CD4+ T cells, activated memory CD4+ T cells and dendritic cells were enriched in the low-risk group compared with the high-risk group. An increased density of CD4+ T cells is considered a marker of good prognosis, which may explain the better prognosis of the low-risk group (41, 42). Furthermore, the proportion of macrophages was significantly increased in the high-risk group compared with the low-risk group. Increased macrophages are related to a poor prognosis because of their pivotal role in innate immunity (43, 44). Stromal cells and immune cells are important components of the tumor microenvironment, and stromal and immune scores are relevant to the prognosis of COAD (45). Through the ESTIMATE algorithm, we estimated the stromal score, immune score, and ESTIMATE score of the high- and low-risk groups based on the ITH-related prognostic signature. Our results showed that the stromal score, immune score, and ESTIMATE score of the high-risk group were significantly elevated compared with those of the low-risk group. This suggests that ITH may participate in the tumor microenvironment, thereby affecting the occurrence and development of tumors. In summary, the ITH-related prognostic signature was significantly associated with immune cell infiltration and may be helpful for immunotherapy and targeted therapy of COAD patients.

Chemotherapy remains one of the main modalities for treating COAD (46, 47). However, accumulating evidence suggests that chemotherapy resistance is a major cause of increased recurrence and mortality in COAD patients, preventing patients from benefiting from standard treatment regimens (48, 49). In this study, we performed chemotherapeutic drug sensitivity analysis for patients in the high- and low-risk groups based on the GDSC database. The patients in the high-risk group were more sensitive to bleomycin, gefitinib, nilotinib, pazopanib, rapamycin, and vinblastine. Loss of ErbB3 expression in poorly differentiated colorectal cancer cells increases sensitivity to gefitinib (50). Nilotinib may benefit colorectal cancer patients by targeting DDR1 kinase (51). In contrast, the patients in the low-risk group were more sensitive to sorafenib and temsirolimus. Sorafenib can be used to treat colorectal cancers resistant to irinotecan-based chemotherapy by inhibiting the ABCG2 drug efflux pump (52). Temsirolimus enhances the efficacy of cetuximab in the treatment of colon cancer by targeting CIP2A expression (53). Additionally, we predicted five small-molecule drugs that could potentially be used to treat COAD patients based on the DEGs between the high- and low-risk groups. AS-605240 is a PI3Kγ selective inhibitor that attenuates chemotherapy-induced cardiotoxicity and breast tumor growth (54). The MAP kinase inhibitor AS-703026 in combination with everolimus can induce apoptosis of melanoma cells (55). Baeomycesic acid exerts antiproliferative effects on twelve different human cancer cell lines (56). The GABA(B) receptor-positive allosteric modulator CGP-7930 can exert antidepressant and anxiolytic effects in animal models (57). As a selective α2-adrenoceptor antagonist, RX 821002 prevents mustard oil-induced ectopic suppression of reflexes by blocking α2-adrenoceptors (58). Here, we predicted five potential small-molecule compounds for COAD that may contribute to the development of new diagnosis and treatment plans.

It is important to note the limitations of the present study. First, we only constructed and validated the signature using retrospective data from the TCGA database, and a multicenter and large-scale prospective study should be carried out to explore its clinical value in the future. Second, further *in vivo* studies are required to explore the five selected genes in the development of COAD. Finally, the extensive association of the signature with multiple drugs warrants our focus on screening potential therapeutic drugs.

## Conclusion

In summary, we constructed a novel ITH-related prognostic signature that can be used to accurately predict survival time in COAD patients. A nomogram integrating clinical factors and the risk score can serve as a rapid initial diagnostic tool for predicting the OS of COAD patients. Furthermore, our signature has implications for the tumor microenvironment and chemotherapy response, which may have valuable clinical significance for improving the effectiveness of treatments, guiding treatment decisions, and saving medical resources.

## Data Availability Statement

The datasets presented in this study can be found in online repositories. The names of the repository/repositories and accession number(s) can be found in the article/Supplementary Material.

## Author Contributions

YX designed the study. CL performed graphing and writing. DL performed data analysis. FW, JuX, and FZ performed literature search. YL, HW, and JR were responsible for language revisions. JiX, JW, and RZ helped modify articles and supervised the study. All authors reviewed the manuscript. All authors contributed to the article and approved the submitted version.

## Funding

This research was funded by the National Natural Science Foundation of China (Nos. 81760105, 82060108, 81970502, and 81460115), the National Key Research and Development Program of China (2016YFC1302201), Youth Project of Jiangxi Province Educational Department (GJJ170124), the Science and Technology Projects of Jiangxi Province (Nos. 20161BBG70113 and 20201ZDG02007), and Leading Talent Training Plan of the Ganpo Outstanding Talents 555 Project of Jiangxi Province (2010-3-61).

## Conflict of Interest

The authors declare that the research was conducted in the absence of any commercial or financial relationships that could be construed as a potential conflict of interest.

## Publisher's Note

All claims expressed in this article are solely those of the authors and do not necessarily represent those of their affiliated organizations, or those of the publisher, the editors and the reviewers. Any product that may be evaluated in this article, or claim that may be made by its manufacturer, is not guaranteed or endorsed by the publisher.

## Supplementary Material

Supplementary Figure S1 Analysis workflow of this study.

Supplementary Figure S2 Validation of the ITH-related prognostic signature in the entire cohort. **(A)** The distribution plot of the risk score (upper), survival time (middle), and heatmap for the expression of five genes (below). **(B)** KM survival analysis of OS between the high- and low-risk groups. **(C)** ROC curve analysis of the ITH-related prognostic signature for predicting the 1-, 3-, and 5-year OS of COAD patients.

Supplementary Figure S3(A,B) Univariate Cox regression analysis **(A)** and multivariate Cox regression analysis **(B)** of the risk score and clinical characteristics. **(C)** DCA of the nomogram.
