# Supplementary figures and images for: An Intratumor Heterogeneity-Related Signature for Predicting Prognosis, Immune Landscape, and Chemotherapy Response in Colon Adenocarcinoma
Source: Front Med (Lausanne). 2022 Jul 7;9:925661. doi: 10.3389/fmed.2022.925661 (PMC9302538; doi:10.3389/fmed.2022.925661)

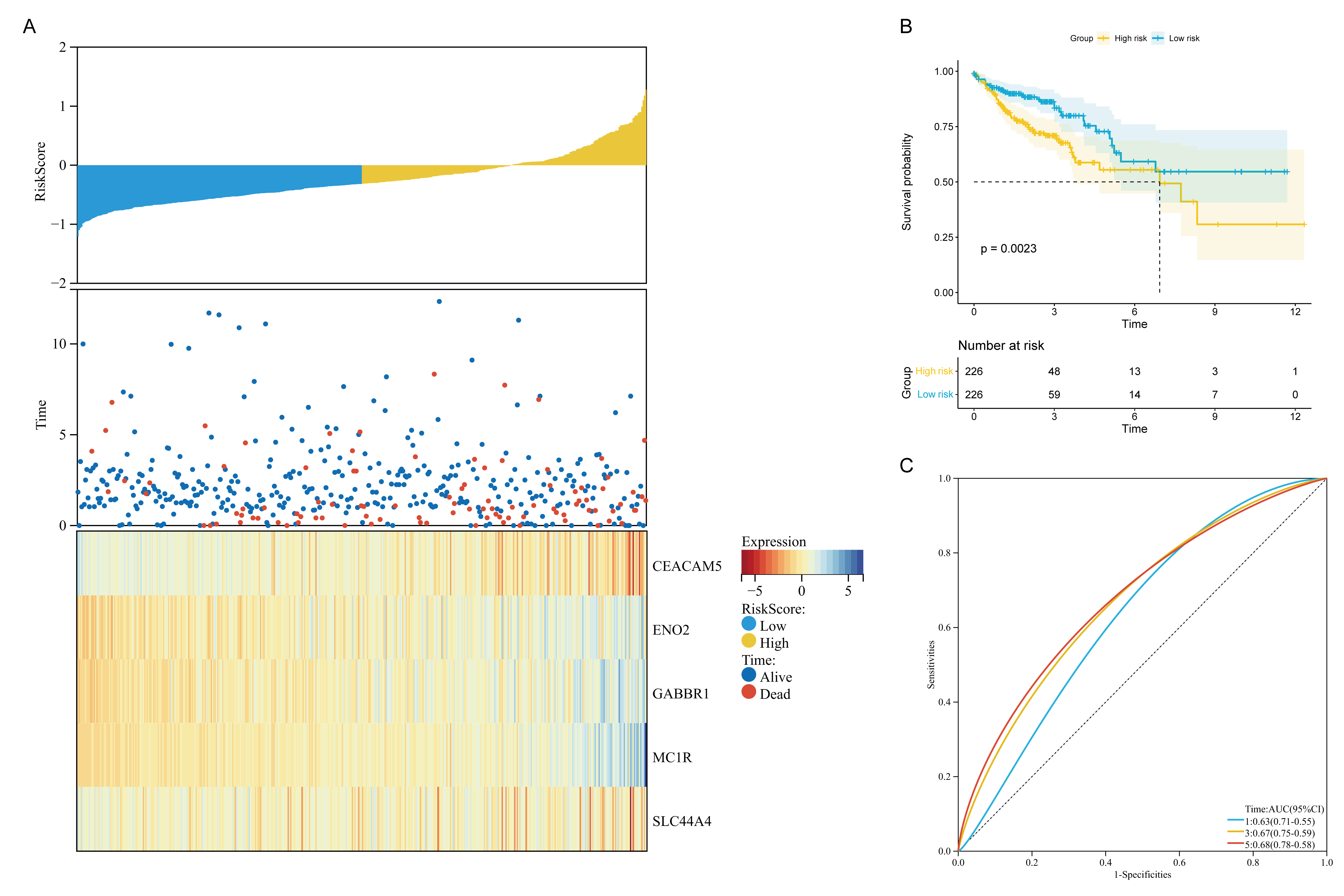

Supplement: Supplementary Figure S2 — Validation of the ITH-related prognostic signature in the entire cohort. (A) The distribution plot of the risk score (upper), survival time (middle), and heatmap for the expression of five genes (below). (B) KM survival analysis of OS between the high- and low-risk groups. (C) ROC curve analysis of the ITH-related prognostic signature for predicting the 1-, 3-, and 5-year OS of COAD patients. [file Image_2.tif]

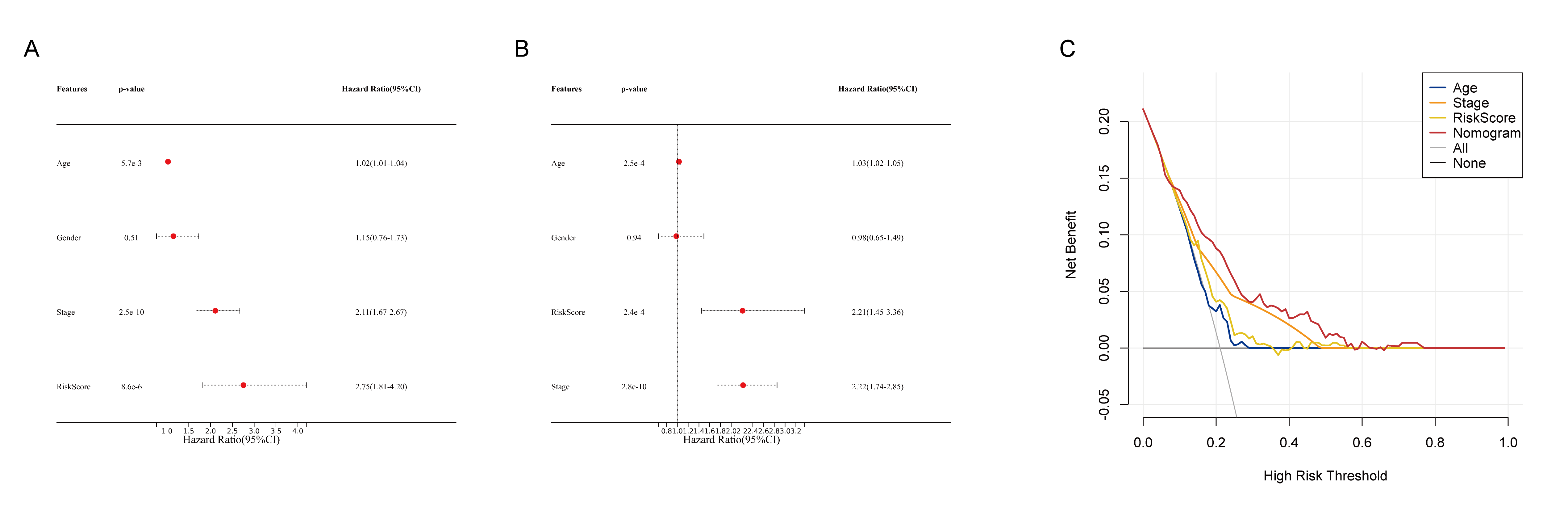

Supplement: Supplementary Figure S3(A,B) — Univariate Cox regression analysis (A) and multivariate Cox regression analysis (B) of the risk score and clinical characteristics. (C) DCA of the nomogram. [file Image_3.tif]
